# Langerhans cell histiocytosis: another cause of a fluid–fluid level within an appendicular bony lesion

**DOI:** 10.1259/bjrcr.20150408

**Published:** 2016-12-01

**Authors:** Venkata Rama Krishna Varanasi, May Ying Leong, Ah Moy Tan, Wen Quan Derrick Lian, Eu Leong Harvey James Teo

**Affiliations:** ^1^ Department of Diagnostic and Interventional Imaging, KK Women’s and Children’s Hospital, Singapore, Singapore; ^2^ Department of Pathology and Laboratory Medicine, KK Women’s and Children’s Hospital, Singapore, Singapore; ^3^ Department of Pediatrics/Division of Hematology and Oncology, KK Women’s and Children’s Hospital, Singapore, Singapore

## Abstract

We report a case of Langerhans cell histiocytosis (LCH) occurring in the pelvis of a 2-year 11-month-old female with fluid–fluid level seen on MRI. Aspiration of the fluid during biopsy showed it to be blood with a few inflammatory cells and eosinophils. Tissue obtained during the biopsy confirmed the diagnosis to be LCH. While fluid–fluid levels have been infrequently encountered in skull lesions due to LCH, they have yet to be reported in lesions of the appendicular skeleton. The aim of this report is to familiarize radiologists with the fact that fluid–fluid levels can occur in LCH of the appendicular skeleton in children.

## Summary

We report a case of Langerhans cell histiocytosis (LCH) occurring in the pelvis of a 2-year 11-month-old female with fluid–fluid level seen on MRI. Aspiration of the fluid during biopsy showed it to be blood with a few inflammatory cells and eosinophils. Tissue obtained during the biopsy confirmed the diagnosis to be LCH. While fluid–fluid levels have been infrequently encountered in skull lesions due to LCH, they have yet to be reported in lesions of the appendicular skeleton. The aim of this report is to familiarize radiologists with the fact fluid–fluid levels can occur in LCH of the appendicular skeleton in children.

## Clinical presentation

A 2-year 11-month-old female presented to our hospital with left hip pain. Laboratory tests did not reveal any significant finding and the inflammatory markers were not elevated.

## Imaging findings

**Figure 1.**
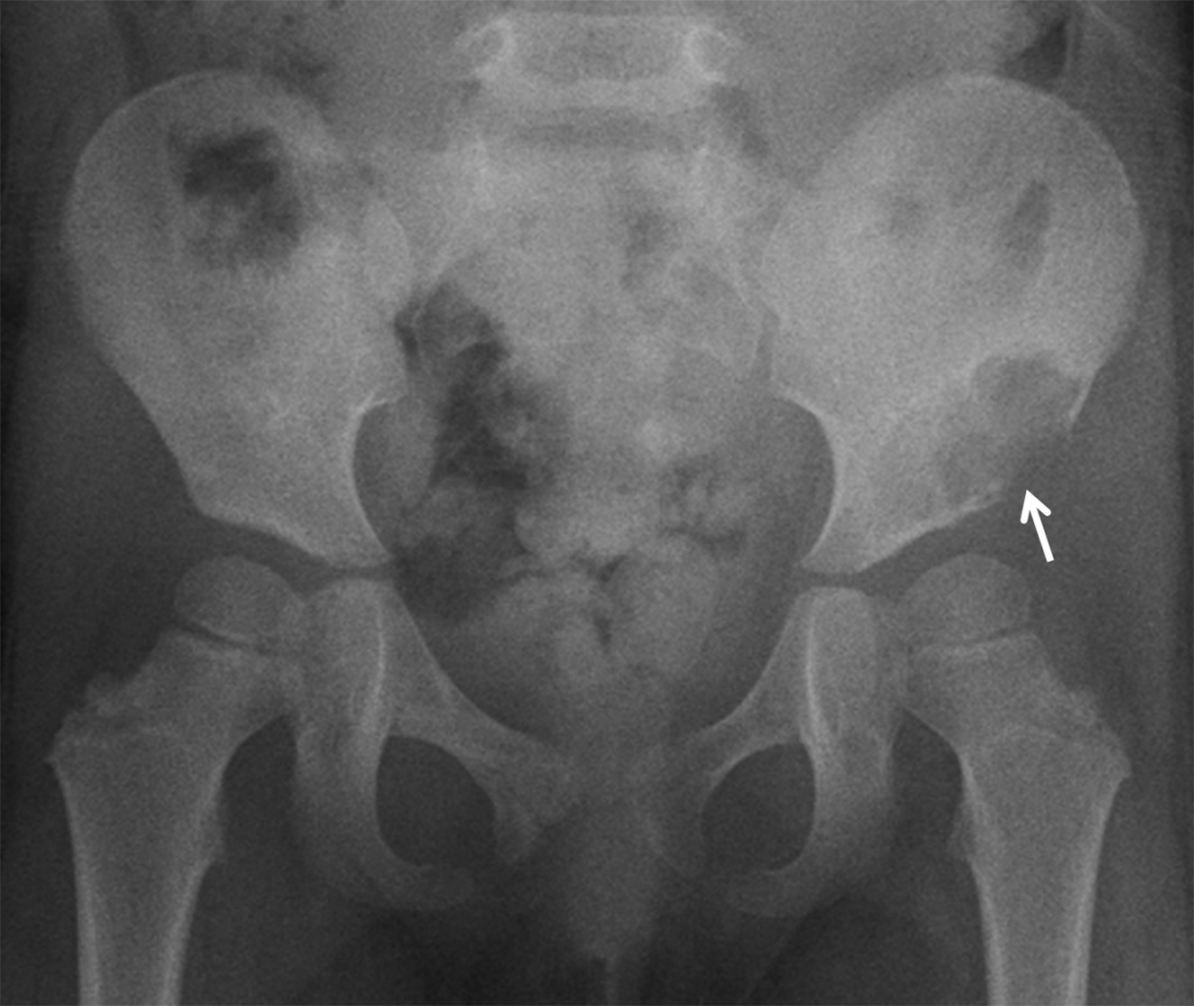
Plain radiograph of the pelvis demonstrates an osteolytic lesion in the left iliac wing (arrow).

**Figure 2.**
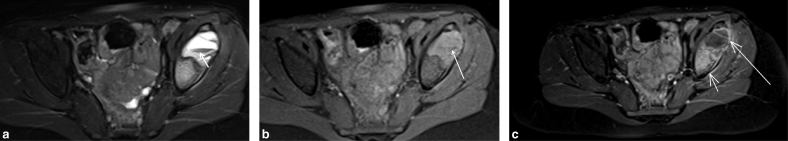
MRI of the pelvis demonstrating the fluid–fluid level. (a) *T*_2_ weighted fat-saturated axial image demonstrates the fluid–fluid level (arrow) with low signal in its dependent portion, suggestive of blood products. (b) *T*_1_ weighted fat-saturated axial image faintly demonstrates the fluid–fluid level (arrow). (c) Post-contrast *T*_1_ weighted fat-saturated axial image demonstrates a linear enhancing septum within the osteolytic lesion (long arrow). Enhancement of the bone (short arrow) adjacent to the osteolytic lesion with fluid–fluid level is noteworthy.

Plain radiographs revealed an osteolytic lesion in the left iliac wing (Figure 1). The lesion was well defined in some areas but also showed cortical destruction with no discernible periosteal reaction. Fat-saturated *T*_2_ weighted images showed fluid–fluid level within the lesion (Figure 2a). The non-dependent part of the fluid was similar to water and the dependent portion was low in signal intensity, suggestive of blood products. Fat-saturated *T*_1_ weighted MRI showed the lesion to be well defined and isointense to muscle. A faint fluid–fluid level was noted within the lesion (Figure 2b). After the administration of intravenous gadolinium (gadopentetate dimeglumine 0.1 mmol kg^–1^), the lesion showed peripheral enhancement and an enhancing internal septa was identified within the lesion (Figure 2c). There was also enhancement of the bone surrounding the lesion. The fluid–fluid level within the lesion was unchanged in appearance. The surrounding bone that enhanced on the *T*_1_ weighted images was high in signal intensity on the fat-saturated *T*_2_ weighted images, possibly owing to bony inflammation. Periosteal reaction was seen in both sequences. Another smaller lesion was noted within the right superior pubic ramus, with mild enhancement on post-contrast images. This lesion appeared solid and did not have a fluid–fluid level.

**Figure 3.**
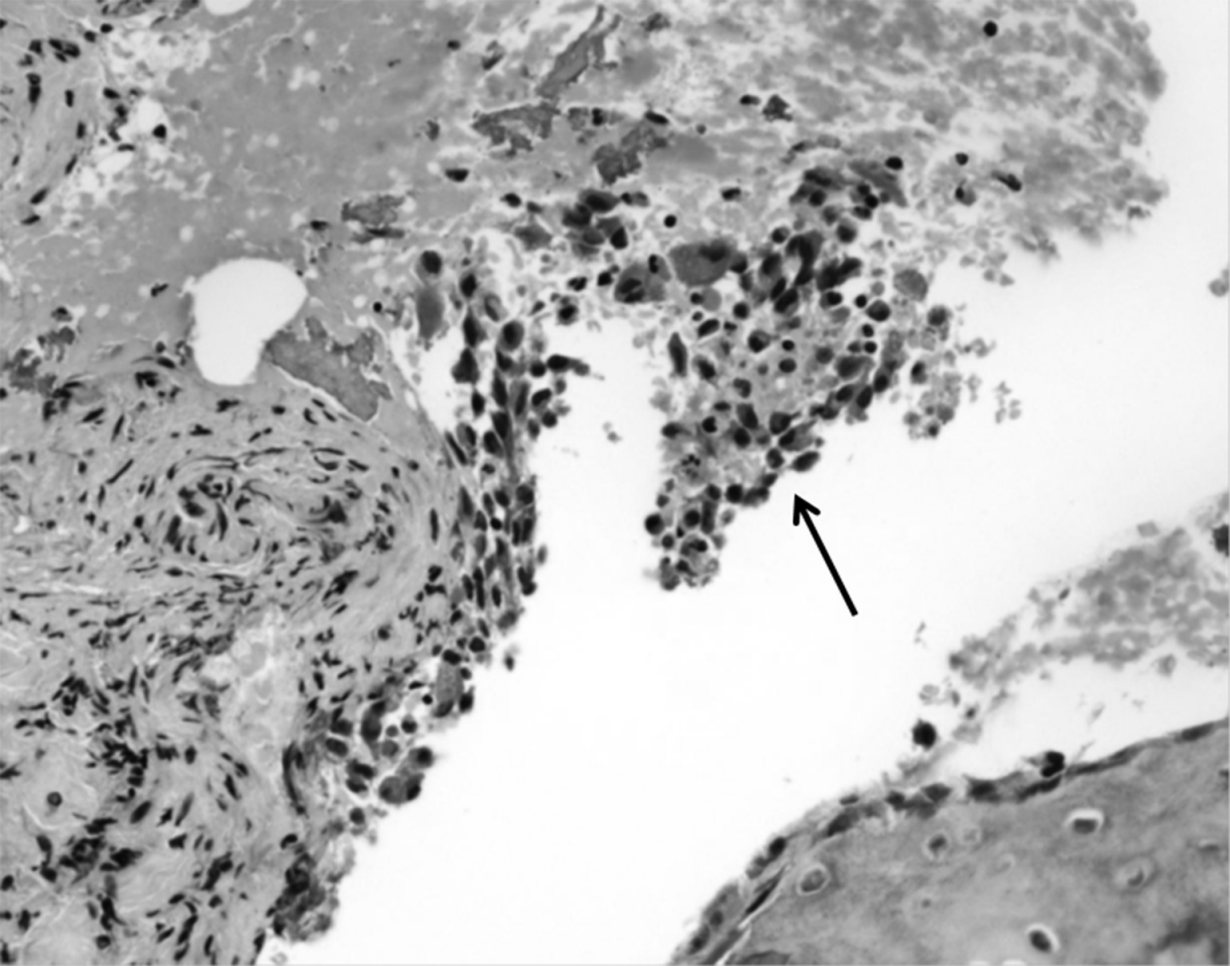
Biopsy from the left iliac bone lesion shows clusters of histiocytic cells (arrow) featuring moderate amounts of eosinophilic cytoplasm and reniform nuclei associated with a few eosinophils (haematoxylin and eosin, magnification ×400). These clusters of histiocytic cells show reactivity for CD1a and langerin.

CT-guided aspiration of fluid contents followed by biopsy of the larger left iliac bone lesion was performed. Blood with a few inflammatory cells and eosinophils was aspirated. The biopsy (Figure 3) and immunohistochemistry confirmed it as LCH.

A subsequent skeletal survey revealed another lesion in the T11 vertebral body. The patient was treated with intravenous vinblastine and oral prednisolone for 6 weeks and is currently well on regular follow-up.

## Discussion

Fluid–fluid levels are characteristically described in aneurysmal bone cysts^1^ but are found in a wide range of bone lesions and are thus a non-specific finding.^2–5^ Fluid–fluid levels have been described in LCH lesions involving the calvarium but not of the appendicular skeleton.^6^This is the first reported case of a fluid–fluid level occurring in a case of LCH of the appendicular skeleton. The occurrence of fluid–fluid level is believed to be due to intratumoral haemorrhage.^7^ This case is important as it further highlights the non-specificity of fluid–fluid levels in osteolytic lesions and LCH should still be included in the differential diagnoses of these lesions in children.

## Learning points

1. The aim of this report is to familiarize radiologists with the fact that fluid–fluid levels can occur in LCH of the appendicular skeleton in children.
2. This case also further highlights the non-specificity of fluid–fluid level in osteolytic lesions.

## Consent

Written informed consent was obtained from the patient’s parents for publication of this case report, including accompanying images.
